# A Real-Time Clinical Endoscopic System for Intraluminal, Multiplexed Imaging of Surface-Enhanced Raman Scattering Nanoparticles

**DOI:** 10.1371/journal.pone.0123185

**Published:** 2015-04-29

**Authors:** Ellis Garai, Steven Sensarn, Cristina L. Zavaleta, Nathan O. Loewke, Stephan Rogalla, Michael J. Mandella, Stephen A. Felt, Shai Friedland, Jonathan T. C. Liu, Sanjiv S. Gambhir, Christopher H. Contag

**Affiliations:** 1 Department of Mechanical Engineering, Stanford University, Stanford, California, United States of America; 2 Department of Radiology, Stanford University, Stanford, California, United States of America; 3 Department of Pediatrics, Stanford University, Stanford, California, United States of America; 4 Departments of Bioengineering and Materials Science and Engineering, Stanford University, Stanford, California, United States of America; 5 Department of Microbiology & Immunology, Stanford University, Stanford, California, United States of America; 6 Division of Gastroenterology and Hepatology, Stanford University, Stanford, California, United States of America; 7 Molecular Imaging Program at Stanford (MIPS), Stanford University, Stanford, California, United States of America; 8 Comparative Medicine, Stanford University, Stanford, California, United States of America; 9 Department of Mechanical Engineering, University of Washington, Seattle, Washington, United States of America; 10 Canary Center at Stanford for Cancer Early Detection, Stanford University, Stanford, California, United States of America; 11 Department of Electrical Engineering, Stanford University, Stanford, California, United States of America; City University of Hong Kong, HONG KONG

## Abstract

The detection of biomarker-targeting surface-enhanced Raman scattering (SERS) nanoparticles (NPs) in the human gastrointestinal tract has the potential to improve early cancer detection; however, a clinically relevant device with rapid Raman-imaging capability has not been described. Here we report the design and *in vivo* demonstration of a miniature, non-contact, opto-electro-mechanical Raman device as an accessory to clinical endoscopes that can provide multiplexed molecular data via a panel of SERS NPs. This device enables rapid circumferential scanning of topologically complex luminal surfaces of hollow organs (e.g., colon and esophagus) and produces quantitative images of the relative concentrations of SERS NPs that are present. Human and swine studies have demonstrated the speed and simplicity of this technique. This approach also offers unparalleled multiplexing capabilities by simultaneously detecting the unique spectral fingerprints of multiple SERS NPs. Therefore, this new screening strategy has the potential to improve diagnosis and to guide therapy by enabling sensitive quantitative molecular detection of small and otherwise hard-to-detect lesions in the context of white-light endoscopy.

## Introduction

Endoscopy is currently the gold standard for cancer screening in the gastrointestinal (GI) tract. This white-light imaging modality enables clinicians to inspect the esophagus, stomach, and colon to detect lesions such as adenomas and carcinomas. GI endoscopy relies upon an examiner’s ability to detect gross anomalies on an otherwise healthy luminal surface. In the colon, many precancerous lesions present as polyps that protrude from the surface by more than 2 mm and are reliably detected by visual inspection. However, the detection of precancerous lesions in the esophagus and stomach is considerably more challenging because most of these lesions do not elevate into a polypoid shape. There exists an increasing recognition of the importance of detecting non-polypoid precancerous lesions in the colon and the contribution of missed lesions to the failure of colonoscopy-based cancer prevention programs [1–4].

Endoscopic detection of small polyps and flat lesions in the colon could be improved by adding an imaging modality that is capable of providing functional (i.e., molecular) information in addition to the structural macroscopic information provided by white-light imaging. Incorporating a molecular imaging modality could help clinicians to locate, diagnose, and stage small and otherwise hard-to-detect lesions, as well as to improve the characterization of these lesions.

Raman spectroscopy can be used as a label-free modality providing information about basic chemical bonds (e.g., hydrocarbons or nucleic acids) through the analysis of the nominally weak inelastic scattering of photons by such molecular motifs *in vitro* or in living tissues [5]. Unfortunately, the detection of weak inelastic scattering of photons in tissues (commonly referred to as intrinsic Raman spectroscopy) typically requires long integration times (minutes) and physical contact between the optical probe and the target tissue. However, with surface enhanced Raman scattering (SERS) nanoparticles (NPs) (~120 nm in diameter), the weak inelastic scattering of specific Raman-active molecules can be increased by many orders of magnitude through a plasmonic enhancement effect, thereby allowing SERS NPs to serve as a source of significant Raman signal. This enhancement (by as much as 10^14^) is possible due to the multilayer (metallic core surrounded by a layer of Raman-active molecules) structure of SERS particles [6–8]. An outer layer of silica encapsulates the NPs, preventing unwanted chemical interactions with surrounding tissue, such that the NPs emit a Raman spectrum that is unique and independent of the surrounding environment. Moreover, by varying the Raman active layer, a panel of different SERS NPs types can be created, each emitting a characteristic fingerprint-like spectrum [7,8]. Since each type, or flavor, of NP has a unique and complex spectral signature they can be readily multiplexed. The advantage of multiplexing is that it allows for the simultaneous detection of multiple biomarkers if each flavor of NP preferentially binds to a different protein target. In light of the large variability in the molecular phenotypes of diseases, multiplexed molecular detection has the potential to improve specificity of disease detection. An additional advantage of SERS NPs is that one of the multiplexed NP flavors can be used as a negative control to account for nonspecific sources of contrast such as nonspecific binding, uneven contrast-agent delivery, and varying working distances between the optical device and the sample [9]. These two features, high sensitivity and multiplexing capability, make SERS NPs ideal candidates as tumor-targeted contrast agents.

Our group, as well as others, have demonstrated the tumor targeting capabilities of several types of conjugated Raman nanoparticles in preclinical animal models and with preclinical systems [7,8,10–17]. However, a clinically relevant device for detecting and imaging these tumor targeting SERS NPs has not yet been described, precluding the translation of this potentially significant molecular imaging approach to the clinic. Therefore, we have focused on the development of an entirely new accessory device that capable of intraluminal SERS imaging during GI endoscopy.

We previously described a point-detection device that demonstrated its potential use with SERS NPs as a screening tool for cancers of the esophagus, colon, cervix, or skin in circumstances where small areas of tissue need to be examined. However as a point-detection device, images could only be created on-the-bench by physically moving the sample [9,18], which is not a viable approach for *in-vivo* imaging of large, complex surfaces. In order to comprehensively scan a portion of the GI tract, such as within the colon or esophagus, within a clinically relevant period of time, a new scanning imaging system needed to be developed.

Here, we describe the design, development and testing of a new opto-electro-mechanical device that has the capability to rapidly and systematically scan large tissue surfaces and produce images of structural and multiplexed functional data within the context of the traditional endoscopic-imaging procedure. In addition, we developed software to display the images in a manner that is readily interpretable by the clinician. The data can be displayed as flat 2-D images, or rendered onto a cylindrical surface to depict organs like the colon and esophagus, where the user can rotate the volume renderings and view the data from any direction to further improve interpretation.

The form factor of our imaging device was designed to allow it to be inserted through the accessory channel of a clinical endoscope and to be used in parallel with white-light endoscopy screening. Circumferential scanning could then rapidly assess the binding of functionalized SERS NPs to tumor targets, thus providing multiplexed molecular information from the entire tissue surface. Here we demonstrate that signals from a panel of SERS flavors can be simultaneously collected and processed into quantitative multiplexed images to inform and guide the endoscopist.

A unique feature of this device is that it is a non-contact system, which has been designed and optimized for efficient use over a wide range of clinically relevant working distances. The human colon has an average radius of 25 mm when insufflated [19]. Assuming the endoscope is held near the center axis of the colon, the average working distance between the device and the colon wall is 25 mm. The non-contact feature also enables rapid scanning of topologically complex tissue surfaces such as the GI tract that consists of folds and bends.

The approving committee for the human study is the Stanford University IRB; the IRB protocol ID is 15766. Written informed consent for the study was obtained from all patients. The IRB approved written informed consent as the consent procedure.

## Results

**Fig 1:**
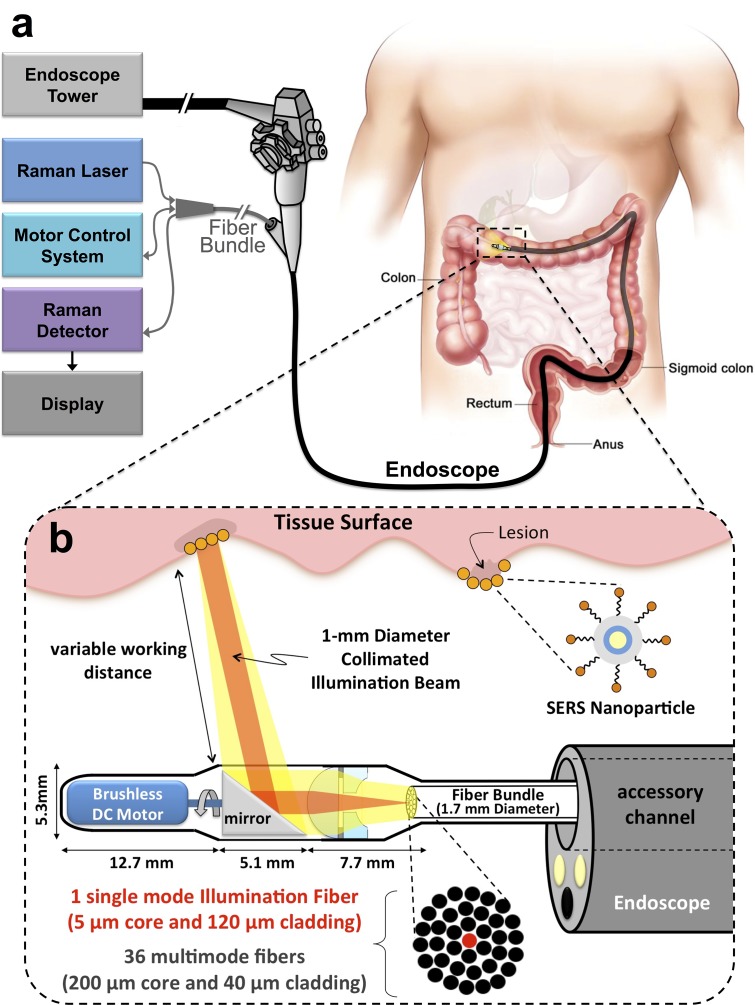
Schematic of Raman-imaging system being used in parallel with white-light endoscopy. (a) The device is designed such that it can be inserted through the accessory channel of a clinical endoscope. As the endoscope is being retracted in the GI tract, the device simultaneously scans the lumen. The collected Raman-scattered light is analyzed, and an image is displayed to the user (also see S1 Video). (b) Expanded schematic of the distal end of the device. The schematic illustrates the position of the device relative to the end of the endoscope. A brushless DC motor that rotates a mirror causing the collimated beam to sweep 360 degrees, enabling luminal imaging of the colon wall. The device is not required to be in contact with the tissue, which is enabled through the use of the collimated illumination beam. A custom, miniature, concentrically segmented, air-spaced doublet lens having a non-reciprocal optical path consists of a plano-convex lens and an adjacent plano-concave lens with a central hole. The doublet lens increases collection efficiency at longer, clinically relevant working distances.

**Fig 2:**
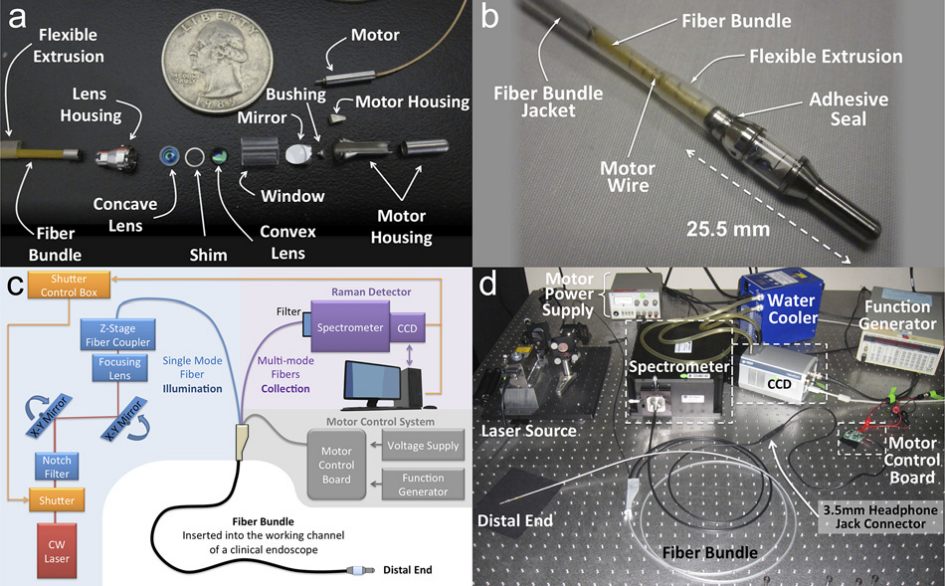
Imaging device and system. (a) Photographs of the components of the distal end of the device adjacent to a quarter (diameter = 24 mm) for scale. All the components shown, less the motor, were custom designed and fabricated for this device. (b) Close-up photograph of the distal end of the fully functional device. The fiber bundle was enclosed and sealed within a flexible extrusion sheath. The window was placed between the scan mirror and the tissue in order to seal the inner mechanisms of the device from fluids in the surrounding environment. The use of a toroidal mirror compensates for beam distortion from the curvature of the glass window in order to maintain a collimated beam. Utilization of a 50-degree inclination angle of the toroidal mirror effectively eliminates back reflections from the window into the fiber bundle detector. (c) System overview. A continuous wave (CW) laser at 785 nm was used and the Raman-scattered light is collected through the multi-mode fibers of the fiber bundle. At the proximal end of the fiber bundle, the multimode fibers are arranged into a vertical array for efficient coupling to the spectrometer. A long-pass filter at the entrance of the spectrometer filters out the illumination light. A function generator signal controls a motor control board to finely tune the rotational speed of the motor to the desired speed of 1 rev/s. (d) Photograph of the completed fully functional system.

**Fig 3:**
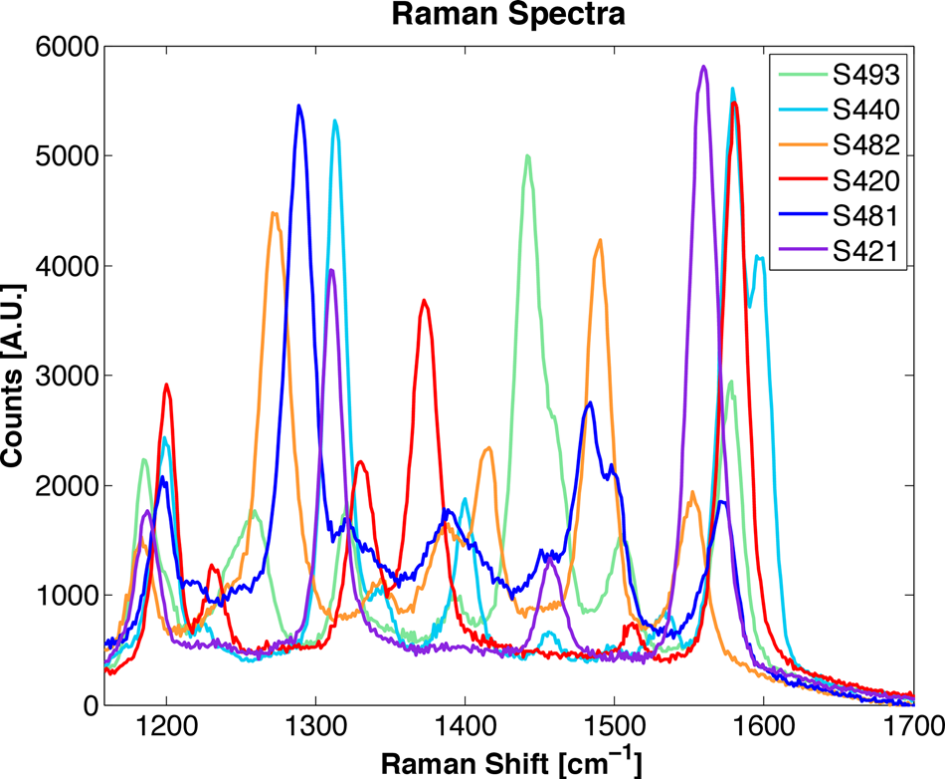
Raman Spectrum of various SERS nanoparticles. The spectrum shown here are for 6 different SERS nanoparticles types, or flavors: S493, S440, S482, S420, S481, and S421. The only difference in the construction of each flavor is the Raman active layer, which results in unique spectra. The units for the x-axis is given in Raman Shift, which can be calculated as follows: Raman Shift = 1/λ_ex_ -1/λ_R_, where λ_ex_ is the excitation wavelength and λ_R_ is the Raman spectrum wavelength. The pump wavelength used in the system described in this manuscript is occurring at 785 nm. Therefore, a Raman Shift of zero corresponds to the pump wavelength.

We have created a small, flexible, fiber-optic-based Raman imaging device, designed for human use, that utilizes circumferential scanning to comprehensively image the lumen of a hollow organ (Figs 1 and 2). By utilizing this device in phantoms of hollow organs and in swine, we demonstrate simultaneous imaging of a panel of SERS NPs in clinically relevant models. Circumferential scanning during retraction of the endoscope by the endoscopist (Fig 1a) and S1 Video) allows mapping of the signal from SERS NPs located on a luminal surface. In order to achieve comprehensive imaging of a hollow organ during endoscopy, a rotating mirror is located between the collimating lens and the tissue and is angled at 50 degrees to provide a radial projection of the collimated beam (Fig 1b). As the scan mirror rotates about its axis, the 1-mm diameter collimated illumination beam sweeps around the device, resulting in a complete 360-degree circumferential scan of the tissue. The scan mirror is actuated with a small (2mm outer diameter) brushless DC motor (P/N: 0206B, Faulhauber) to provide rotational scanning at one revolution per second (Fig 2a–2b) and S1 Video). To compensate for astigmatism on the collimated beam caused by cylindrical lensing due to the window, we use a scan mirror with a compensating toroidal surface profile (see S1 File). In addition, the 50-degree angle of the mirror face redirects back reflections from the glass window away from the signal collection path, which significantly reduces noise in the system by more than 90 dB (please see S1 File for more details). A 785 nm continuous wave (CW) laser diode (iBeam Smart; Toptica Photonics) is used for illumination and is coupled into the single-mode fiber (5 μm mode-field-diameter) located at the center of the fiber bundle. This wavelength was chosen for illumination in order to minimize absorption and scattering from the tissue surrounding tissue. A plano-convex lens (4-mm outer diameter, 4.63-mm focal length) collimates the illumination beam after it passes through a central hole in the adjacent plano-concave lens (Fig 1b). As the laser beam sweeps around the circumference of the organ lumen, the collected signal passes through the window and is redirected by the scan mirror along the optical return path. The return path utilizes both the plano-convex and plano-concave lenses (4-mm outer diameter, 2.2-mm radius of curvature, 1-mm-diameteter central hole), which together form an optical system having a longer effective focal length for signal collection than in the illuminating direction. This results in an improvement in collection efficiency by more than 200% as compared to a single lens system (see S1 File). 36 separate multi-mode fibers (200 μm core diameter, 20 μm cladding) surround the single-mode fiber within the fiber bundle and guide the Raman scatter light from the distal end to the spectrometer at the proximal end. Further details of the proximal end of the system can be found in S1 File. The unique Raman spectra of the SERS nanoparticles used can be seen in Fig 3. Acquired spectra by the system are unmixed using a hybrid algorithm combining least squares and principal component analysis [9]. The algorithm produces weighting factors quantifying the relative amounts of each SERS NP flavor present in the sample, as well as quantifies and corrects for systematic background signal generated within the endoscope and subtle fluctuations in the acquired spectrum associated with outside noise sources.

### Device Sensitivity

To characterize sensitivity, the device was placed at a distance of 25 mm from the surface of a series of wells containing various concentrations of NPs to mimic the nominal working distance of an endoscopic device in a human colon. Staying within maximum permissible exposure (MPE) limits for incident light, the smallest sample of NPs that produces a signal of at least one standard deviation above the mean background is 783,000 NPs (see S1 File for calculation from concentration to number of nanoparticles and S1 File for statistical analysis). Spread over the laser beam diameter, this corresponds to about 240 functionalized NPs per 100 μm^2^ of tissue (the approximate footprint of the exposed cell surface). If 100 μm^2^ is equivalent to the cross-sectional area of approximately 9,000 SERS nanoparticles, then only 2.7% (or ~240 NPs) of the tissue area would need to be covered by NPs. In other words, a minimum of 783,000 nanoparticles would need to lie within the effective area of the collimated laser (see S1 File for calculation), which is only 2.7% of the total illuminated area. Therefore, our device could detect functionalized SERS NPs sparsely bound to cells by utilizing only a few hundred (~240) receptors per cell, which is relevant for clinical use of this device for detecting clinically relevant tumor markers [20].

### Phantom Model

**Fig 4:**
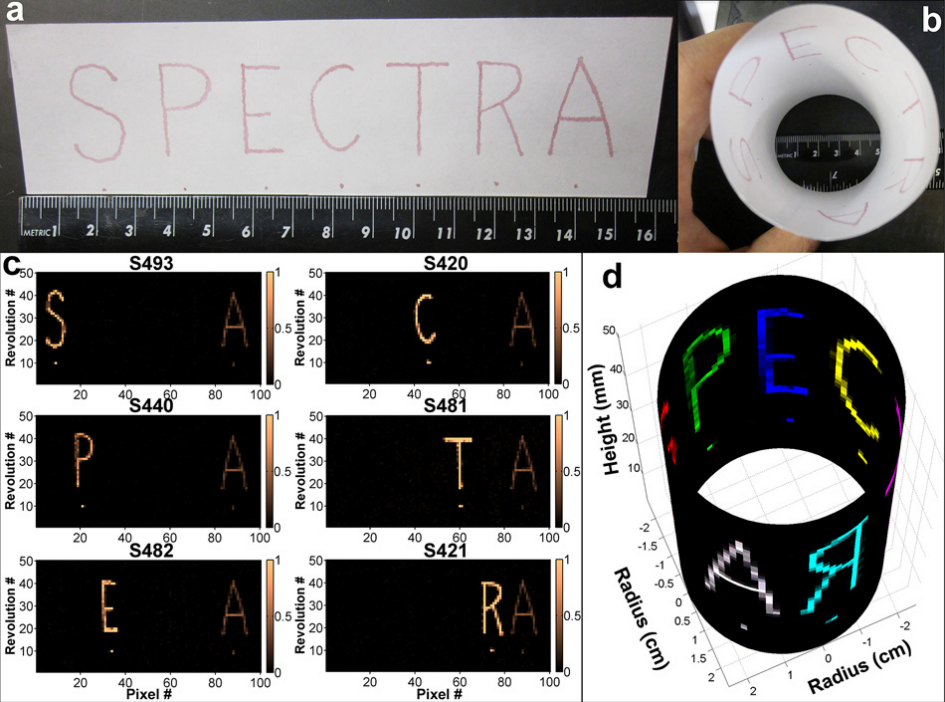
Hollow lumen phantom multiplexing study. (a) Photograph of the paper phantom laid flat. Each letter and the spot beneath was pipetted on to paper using different SERS nanoparticle flavors (‘S’ = S493,‘P’ = S440, ‘E’ = S482, ‘C’ = S420, ‘T’ = S481, ‘R’ = S421). The ‘A’ and spot below were composed of an equal mixture of all six flavors at one-fifth the concentration; thus appearing dimmer than the other letters. The spots under each letter were below 1 mm in diameter, which is less than the size detectable with white-light endoscopy. (b) Photograph of the phantom with a radius set to 25 mm to mimic the average human colon radius. (c) Image of signal intensities of S493, S440, S482, S420, S481, and S421 shown as 2-D images. (d) Cylindrical three-dimensional reconstruction of the data acquired with the device showing a 5-cm segment of the phantom lumen. (see S2 Video). There are a total of 5,000 pixels (50 rev x 100 pix/rev) acquired, which was obtained in a period of 50 seconds (1 rev/s). Each of the SERS flavors was assigned a specific color.

A paper phantom served to demonstrate the multiplexing imaging capabilities, as well as the scanning speed of our device (Fig 4). Moreover, we wanted to demonstrate the real-time imaging capabilities of the system as well. An illumination power of 68 mW and an axial retraction speed of 1 mm/s were used. At a scanning speed of 1 rev/s and an integration time of 10 ms per pixel, 100 pixels per revolution were acquired. A paper phantom with a radius of 25 mm was chosen to mimic the average radius of a human colon. The spots under each letter on the paper phantom were 1 mm in diameter or less, which is smaller than the typical detectable polyp size during white-light endoscopy. See S1 File for more details.

The results are generated in real-time and are displayed both as a 2D image (Fig 4c) and rendered into a cylindrical three-dimensional model (Fig 4d). The images in S2 Video were generated in real-time and each SERS flavor was acquired simultaneously and assigned a unique color. Custom software not only displays the results in real-time, but also allows the user to rotate the cylindrical rendering while the scan is taking place in order to view the results from any direction.

We were able to image the 5-cm long paper phantom in 50 s (Fig 4). While the maximum output from the device was 68 mW, at our integration time of 10 ms, the ANSI and IEC MPE limit (as defined by ANSI-2000 and IEC 60825–1) would allow for an even greater output power of 340 mW for our beam size. By using a more powerful laser such output powers could be achieved to further improve signal-to-noise ratio and sensitivity (see S1 File for more details). To generate the data shown in Fig 4, we used the same unmixing algorithm previously described [9].

### *Ex-vivo* Porcine Colon Multiplexing Study

**Fig 5:**
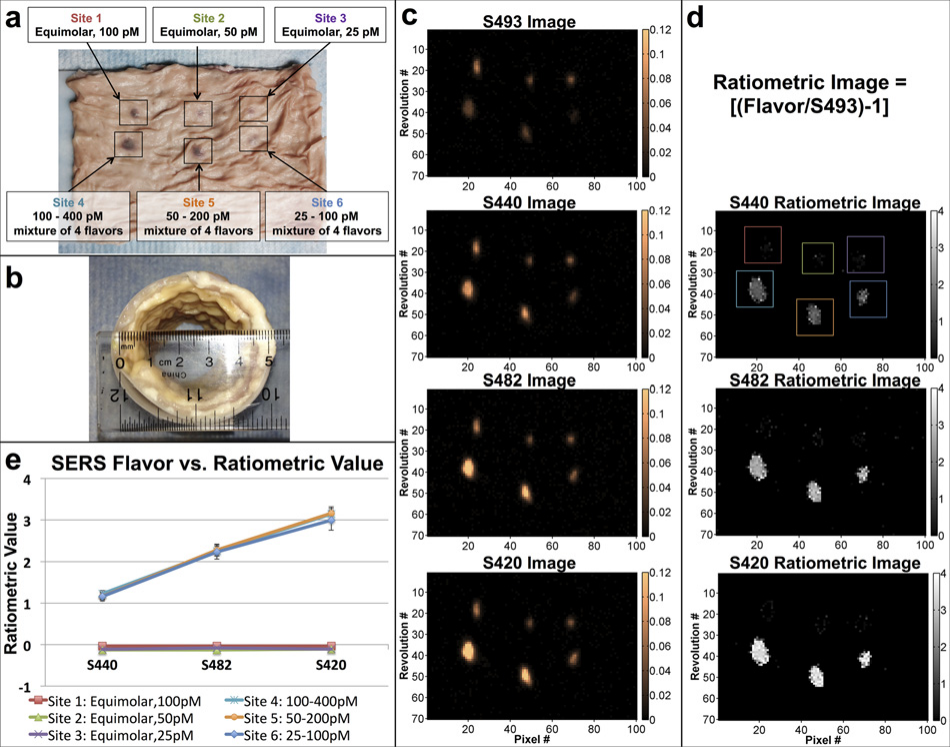
*Ex-vivo* porcine colon multiplexing study. (a) Porcine colon was initially laid flat. Various cocktails of SERS nanoparticles were injected superficially at 6 different sites. (b) The tissue was placed on a flexible sheath and then rolled to re-form the lumen of the colon, which was then scanned with the device. (c) Images of signal intensities of S493, S440, S482, and S420 shown in 2D. (d) Ratiometric images of the tissue samples shown in 2D. ROIs used for analysis are shown. (e) Average of ratiometric values for each of the respective SERS flavors in each tissue-sample ROI from (d). Error bars are standard errors of the mean.

To demonstrate multiplexing capabilities with a tissue model that better resembles the clinical case, excised porcine colon tissue was used. Here, a variety of SERS solutions were injected superficially (Fig 5a) (see S1 File for more details). The signal intensity for the equimolar mixture injections was relatively constant among the four flavors, while at the stepwise mixture injection sites the signal intensity increased linearly for the more concentrated flavors (Fig 5c). This is made apparent by visualizing the ratiometric images, where all flavors are quantified relative to one of the NP flavors: S493 (Fig 5d). Here, S493 is designated as the non-specific control flavor; thus, the quantities displayed in the ratiometric image are: Ratiometric Value = [(Flavor Value)/(S493 Value)– 1]. Values greater than zero indicate locations where the specific SERS flavors are located in greater abundance than the non-specific flavor. Variations in tissue topography and imperfect centering of the device within the colon lumen can also be accounted for and normalized by using this ratiometric algorithm [9]. The quantified results from the 6 injection sites are shown in Fig 5e.

### *In-vivo* Animal Study

**Fig 6:**
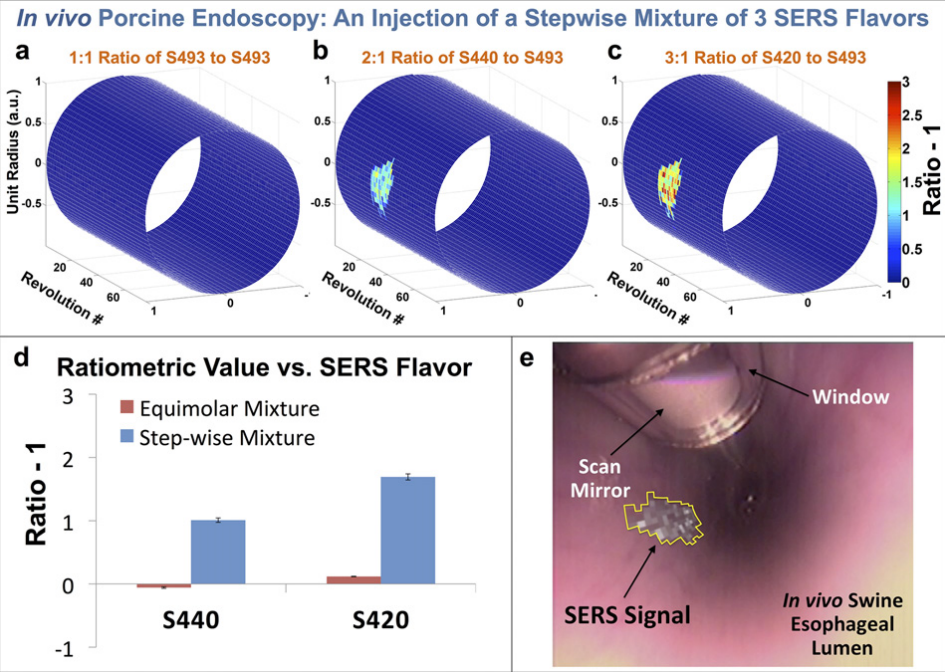
*In vivo* swine endoscopy model multiplexing study. A comparison of images after stepwise injections of a SERS nanoparticle cocktail. Using an endoscope, the injection was made into the esophageal mucosal layer of a fresh intact postmortem pig. The esophagus was then scanned using our device. (a-c) Cylindrical three-dimensional reconstruction of ratiometric signal intensities of S493, S440, and S420 from the stepwise-mixture injection site. A total of 8,000 pixels (80 rev x 100 pix/rev) were acquired in a period 80 seconds (1 rev/s). (a) Ratiometric signal intensity of S493. (b) Ratiometric signal intensity of S440. (c) Ratiometric signal intensity of S420. (d) The center values of each bar in the bar graph are the average of ratiometric values for each of the respective SERS flavors where the value is non-zero (*n* = 123 for S440 equimolar and S420 equimolar, and *n* = 128 for S440 step-wise and S420 step-wise). The error bars are standard errors of the mean. (e) Cylindrical three-dimensional ratiometric image of S420 (from (c)) superimposed onto the white-light endoscope image of the esophagus. The device can be seen in the upper left corner with the scan mirror and window.

In order to demonstrate the detection of NP solutions that cannot be visually identified, two injections of varying mixtures of non-targeted SERS NPs were placed in the submucosal layer of the esophagus of a freshly euthanized pig (see S1 File for more details). The first injection consisted of an equimolar mixture of three flavors in order to mimic random pooling, incomplete wash-out, and nonspecific binding of a panel of NPs. The second injection consisted of a stepwise mixture of three NP flavors of increasing concentrations in order to mimic differential specific binding of various NP flavors. The signal intensity for the equimolar mixture was relatively constant among the three flavors, while that for the stepwise mixture increased linearly with concentration. This is made apparent by visualizing the ratiometric images (Ratio -1), where all flavors are quantified relative to one of the NP flavors: S493 (Fig 6a–6c). For the stepwise mixture injection, the signal from each NP flavor increases in proportion to its actual concentration. The quantified results from the injection sites are shown in Fig 6d. The ratiometric signal from S420 is also overlaid onto a white-light endoscope image to demonstrate a new way to visualize the SERS data in the context of the anatomy (Fig 6e). See S1 File for statistical analysis.

### Colon Phantom Raman Signal with Topography Overlay

**Fig 7:**
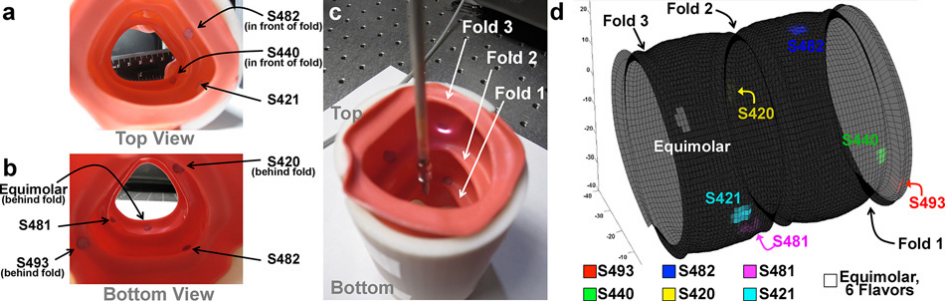
Real-time, multiplexed imaging overlaid onto 3D lumen topography in a colon phantom. (a) Top view of the colon phantom. Location of some the SERS samples are shown, some of which were placed directly in front of a fold (S482 and S481). (b) Bottom view of the colon phantom. Location of some the SERS samples are shown, some of which were placed directly in behind a fold (S493, S420, and the Equimolar sample). The Equimolar sample consists of an equal mixture of all 6 flavors. (c) The phantom measured roughly 8cm tall. The laser can be seen sweeping along the lumen of the surface. (d) Both the topography and Raman signal overlay is being generated simultaneously and in real-time. A total of 6,500 pixels (65 rev x 100 pix/rev) were acquired in a period 65 seconds (1 rev/s). See S3 Video for real-time reconstruction and Raman signal overlay.

The purpose of this study was to demonstrate: 1. Both the Raman signal from the SERS nanoparticles and topography could be acquired simultaneously, 2. The Raman signal can be overlaid with the topological information and displayed in real-time, and 3. Multiplexing of the Raman signal. By displaying the Raman signal overlaid on the topography the user can visually co-register where the Raman signal is originating with relation to the surrounding surface features, most notably the folds. The three-dimensional topography of the colon could be recreated by utilizing the weighting factor for the first principal component of the acquired background signal (see S1 File). This component accounts for most of the variability in the background signal and can be used to quantify its amplitude. The intensity of the background signal, originating from laser light that reflects from the lumen surface back into the device, correlates with the distance from the tissue surface. Samples were placed in front of (Fig 7a) and behind (Fig 7b) folds and were successfully detected with the system (Fig 7d).

### Clinical Use

One of the challenges in designing a device that contains all of the aforementioned functionalities is that it must be able to pass through the accessory channel of the endoscopes. All current clinical endoscopes have a significant bend (~ 30 deg) at the proximal entrance of their accessory channels that limits the rigid length of any inserted device. By minimizing the overall length of the rigid distal end of our imaging device to no more than 25.5 mm (Fig 2b) and transitioning to a flexible fiber bundle, the device was easily inserted through the accessory channel of the endoscope (S4 Video).

While the SERS NPs require USA FDA approval in order to be used in humans, the imaging device alone was used in a patient to analyze the variability of the background signal and the usability of the device. A male patient undergoing routine colonoscopy screening consented to participate in the study and the device was tested in this patient by an experienced endoscopist (S5 Video). Signal was acquired over 30 seconds (1 rev/s, 100 pixels/rev), for a total of 3,000 spectral acquisitions. No intrinsic Raman signal from the tissue was detected, and the system did not pick up spectral peaks from the xenon lamp in the range where SERS NPs emit (see S1 File). Thus, these will not be significant noise sources to interfere with the signals from SERS NPs once the FDA approves them for human use.

**Fig 8:**
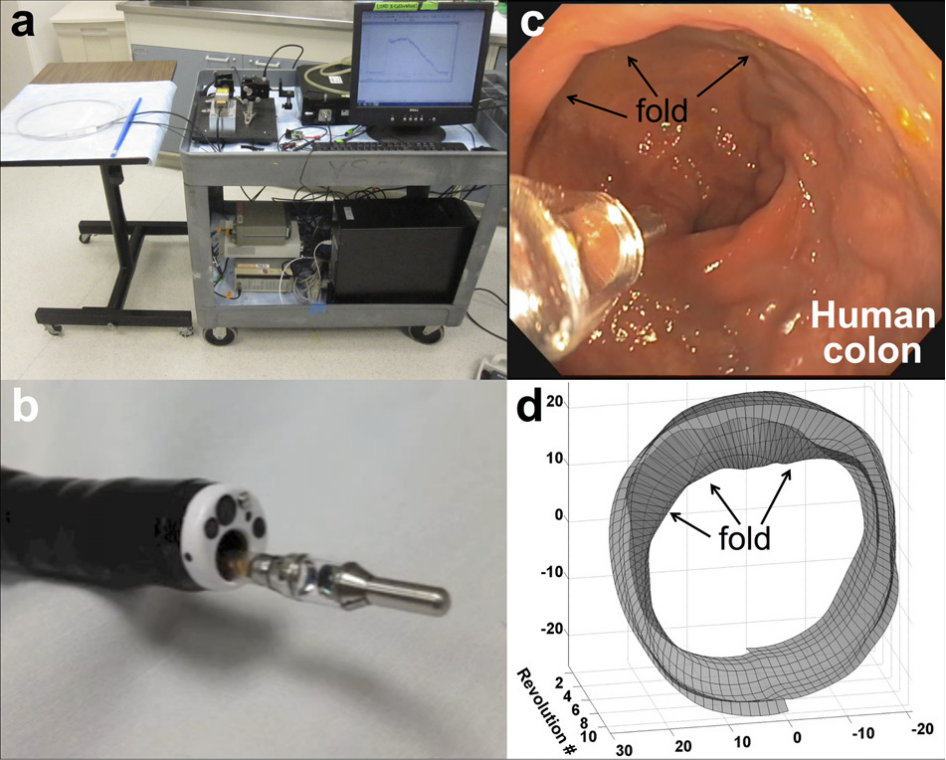
Clinical application and utility of the device in a patient. (a) The imaging system in the endoscopy suite. The entire system can be placed on a single cart measuring 32”×30”×16” before any attempts at improving form factor. (b) The device inserted and exiting from the distal end of the accessory channel of a clinical endoscope (see S4 Video). The device can pass through the sharp bend at the proximal end of the endoscope. The fiber bundle is very flexible and does not compromise the articulation of the distal portion of the endoscope. (c) The device being used in first human clinical study. Video taken from the clinical endoscope shows the device protruding from the accessory channel (see S5 Video). In the video, the illumination beam can be seen scanning the colon wall in a human patient as the endoscope is being retracted. (d) The device was used in a patient to analyze the variability of the background signal and its usability. Analyzing the background data taken with our system, a three-dimensional reconstruction of the topography of the colon wall could be estimated. The video shows a fold in the upper left-hand quadrant that can be visually verified in the white-light image shown by the arrows in (c) (see S6 Video). Ten revolutions of data were acquired for a total of 1,000 pixels (100 pix/rev).

In the *in vivo* human study, the three-dimensional topography of the colon could be recreated by utilizing the weighting factor for the first principal component of the acquired background signal (see Fig 8d and S1 File). The tissue fold in the upper left-hand quadrant in Fig 8d can be visually verified in the white-light image as seen in Fig 8c. These results provide an anatomic reference image on which the molecular data can be plotted.

The limitations of routine colonoscopy in the proximal portion of the colon have been demonstrated in several recent studies [21–23]. With a rotational speed of 1 rev/s and an axial retraction speed of 1 mm/s, approximately 10 min will be required to scan the entire proximal 2 ft of the colon. Because our device can be used simultaneously with the white-light endoscope, it offers the potential for more reliable detection of precancerous lesions without significantly increasing the time of the procedure. Our Raman device is able to scan in the presence of the white light of the endoscope such that both imaging modalities can be performed simultaneously during retraction of the clinical endoscope (S6 Video).

## Discussion

Luminal imaging of multiplexed SERS NPs has the potential to improve the screening and diagnosis of malignant tissues within the GI tract and to guide therapy. Here we demonstrated the scanning ability of our new device in a pilot human clinical test and also showed the ability to image a multiplexed panel of SERS flavors in a swine study. Our device can be deployed through the accessory channel of a clinical endoscope for simultaneous use during routine white-light endoscopy. We have previously demonstrated that the combination of full-spectrum acquisition and our unmixing algorithm can compensate for background signals due to the xenon lamps used in white-light clinical endoscopes [18]. Therefore, as the clinical endoscope is retracted during colonoscopy, our device is able to scan without requiring the endoscope light source to be dimmed or turned off. Finally, because our device is deployed through an endoscope, and used simultaneously during white-light endoscopic imaging, our new imaging modality will add minimal time to the endoscopic screening process.

This is in contrast to autofluoresence and fluorescence endoscopy, which requires the endoscope lights to be turned off (unless near-infrared fluorophore are used). However, practical guidelines for endoscopy require that the clinician not move the endoscope without having a clear view of the investigated organ to prevent injury or perforation. Thus, turning off the light of the endoscope increases the risk of complications as well as the procedural time and the duration in which patient is sedated. Also, fluorescence endomicroscopes like the Cellvizio (Mauna Kea Technologies) provide cellular-resolution images, but at the expense of a large field-of-view (since the space bandwidth product must remain constant). This limits their use to localized, and often predetermined, areas of interest rather than for screening purposes; thus, the ability to survey large areas in a short time frame is not possible.

There is always a general tradeoff between resolution and field-of-view when designing optical systems. Although the resolution and image quality are lower in our approach than others, such as fluorescence microendoscopy, the advantage is that the user is able to scan clinically relevant surface areas quickly and conveniently making it suitable as a screening tool. The use of SERS NPs compensates, in part, for this lower resolution by providing intense signals that enable detection of relatively small lesions as well as enabling multiplexed molecular imaging.

The SERS imaging strategy we propose has additional unique advantages. The complex narrow-band Raman spectra of the SERS NPs allows for simultaneous multiplexed detection using a single excitation wavelength. Although multiplexing can be achieved using fluorophores, their broad emission spectra limit the number of fluorophores that can be unmixed reliably, especially when a single excitation wavelength is used. Also, by measuring the entire spectrum with each acquisition and unmixing with our least-squares and PCA algorithm [9], our system can be used for ratiometric quantification of NPs. This allows for an internal control (since one flavor can be non-specific) and insensitivity to variations in working distance that may be introduced by the clinician or that may occur due to the normal peristaltic action of the organ [9].

The enhanced Raman effect can occur at any frequency of incident light within the broad plasmon resonance of the NP (unlike fluorophores which have specific and comparably narrow absorption peaks that vary with each fluorophore). Thus, we were free to choose the illumination wavelength of our system to be 785 nm, in the near-infrared window where absorption from tissue, water, and blood is minimal [24], allowing us to maximize the sensitivity of our system. Furthermore, the overall wavelength range covered by the SERS NP spectra is constant for all flavors of NPs used in this study (approximately 1100–1600 cm^-1^ Stokes shift from the laser wavelength); this means that the absorption and scattering properties of the tissue affect the detection of all SERS flavors equally. This serves to increase the robustness of ratiometric imaging using SERS NPs as compared to fluorophores. In comparison, the efficient excitation of multiple fluorophores would require more complex illumination, consisting of multiple wavelengths, and multiple filters; thus precluding the sole utilization of the near-infrared transparency window.

An additional advantage of SERS NPs is that they do not photobleach, which is a limitation of quantum dots and fluorophore-based endoscopy. SERS NPs also have the potential to exhibit higher avidity than dyes and labeled reagents since a single NP can bind to multiple receptors. Lastly, in contrast to fluorophores, these SERS NPs emit a uniformly bright signal across the different SERS NP flavors (Fig 4c); plotted on a linear scale). Furthermore, we are developing the next generation SERS NPs with increased Raman signal intensity (for improved sensitivity) and biomarker-targeted functionality.

The non-contact feature of the endoscopic imaging device developed in this study allows the user to scan large, topologically complex surfaces much faster than devices requiring tissue contact. With a rotational speed of 1 rev/s and an axial retraction speed of 1 mm/s, approximately 10 min will be required to scan the entire proximal 2 ft of the colon, which can be performed in parallel with the conventional endoscopy screening. In addition, because the illumination laser is collimated, a constant optical power density can be reliably kept below MPE limits, and resolution is independent of working distance. Topical application of the SERS NPs within the colon lumen, as opposed to intravenous injection, can be performed to potentially minimize toxicity to the patient, as demonstrated in mice [25].

Luminal imaging of multiplexed SERS NPs is a platform technology for rapid functional imaging, which has the potential to distinguish various stages of malignancy and different cancer types in areas of the body accessible to endoscopy, such as the colon, esophagus and stomach, and adapted for use in the oral cavity, cervix, and bladder.

## Supporting Information

S1 Video 3D rendered animation. 3D rendered animation illustrating the illumination beam (red) and collected light (yellow) as well as all the assembly of all inner components. Also shown is the scanning of the beam as the device is deployed through the accessory channel of an endoscope scanning the luminal wall of the colon. Bends and folds of the colon are also depicted in the animation.
(MOV)

S2 Video Real-time, multiplexed imaging in a lumen model. The left frame shows the device scanning the paper phantom as it is being axially retracted. The center and right frames show two real-time displayed representations of the SERS signals as they are being acquired. The signals from all the SERS flavors are being acquired simultaneously and displayed in real-time both as a 2D image (center) and cylindrical three-dimensional model (right). Each of the SERS flavors was assigned a specific color. The right frame of the video, you will see that the image is being periodically rotated about the z-axis. The user is able to rotate the cylindrical three-dimensional rendering at any point during the scan in order to view the results from any direction.
(MOV)

S3 Video Real-time, multiplexed imaging overlaid onto 3D lumen topography in a colon phantom. The white light video on the left shows the phantom and the axial retraction of the device. The beam can be seen sweeping along the lumen surface and the video on the right is being generated in real-time. The signals from all the SERS flavors as well as the topography information are being acquired simultaneously and displayed in real-time. The data is initially displayed from an intraluminal view which is a similar view obtained from white the white light endoscope, but is easily rotated by the user.
(MOV)

S4 Video Device being inserted into a clinical endoscope. (MOV)

S5 Video Device being used in first human clinical study as taken from a clinical endoscope. The illumination beam can be seen scanning the colon wall in a human patient as the endoscope is being retracted.
(MOV)

S6 Video Three-dimensional reconstruction of the topography of the colon wall. (MOV)

S1 File Methods and Statistical Analysis. Further details regarding the device fabrication as well as the methods used for the experimental setup and statistical analysis can be found here.
(DOCX)
