# Supplementary material for: A Real-Time Clinical Endoscopic System for Intraluminal, Multiplexed Imaging of Surface-Enhanced Raman Scattering Nanoparticles
Source: PLoS One. 2015 Apr 29;10(4):e0123185. doi: 10.1371/journal.pone.0123185 (PMC4414592; doi:10.1371/journal.pone.0123185)
Supplement: S1 File — Further details regarding the device fabrication as well as the methods used for the experimental setup and statistical analysis can be found here. (DOCX) [file pone.0123185.s007.docx]

**Supporting Information: Methods**

The approving committee is the Stanford University IRB. The IRB protocol ID is 15766. Written informed consent for the study was obtained from all patients. The IRB approved written informed consent as the consent procedure.

1. **System Design**

The proximal end of the system consists of the following components. A 785 nm laser diode (iBeam Smart; Toptica Photonics) is used to coupled into the single-mode illumination fiber (5 μm mode-field-diameter) and the illumination beam of the system has an overall maximum output of 68mW. 36 separate multi-mode light-collection fibers (200 μm core diameter, 20 μm cladding) allow maximum flexibility of the fiber bundle, as previously described (1). This flexibility is needed for use in fully articulating clinical endoscopes (**S4 Video**). At the proximal end of the fiber bundle, the multi-mode collection fibers are separated from the single-mode illumination fiber and arranged into a linear array to optimize coupling to the entrance slit of a custom spectrometer, dispersing the wavelengths of Raman-scattered light onto the CCD sensor array (DU920P-BR-DD; Andor Technologies), similar to the earlier device (1). The camera readout procedure was set to perform full vertical binning of the sensor in order to quantify the spectral intensity of the Raman signal from all collection fibers at each wavelength (given in units of counts). Acquired spectra are unmixed using a hybrid algorithm combining least squares and principal component analysis (2). The algorithm produces weighting factors quantifying the relative amounts of each SERS NP flavor present in the sample, as well as quantifies and corrects for systematic background signal generated within the endoscope and subtle fluctuations in the acquired spectrum associated with outside noise sources. Our system is capable of filtering out the noise resulting from the white-light source of the endoscope (1), allowing the device to be used simultaneously with conventional white-light endoscopy.

The motor control system consists of a circuit board (P/N: BLD 05002 S, Faulhauber) powered by a DC voltage supply where a function generator is used to finely tune the rotation speed of the motor drive shaft (**Figs. 2(c)** and **2(d)**). The motor (P/N: 0206…B, Faulhauber) operates at a speed of 1 rev/s and is calibrated only once during the initial set-up using a photodetector placed at a fixed position in the path of the illumination beam. The service life of these motors is only limited by the internal bearings. The motor itself has a diameter of 1.9 mm, a length of 5.5 mm, and weighs only 0.09 grams. The motor is rated for a maximum continuous torque of 0.012 mNm and speeds up to 100,000 RPM; however, given that the mirror is the only thing mounted on the motor drive shaft we are well within the maximum specifications of the motor.

In the distal end of the device, both lenses have a deep-V anti-reflection coating centered at 785 nm and work together to collect the Raman scattered light into a multi-mode fiber bundle (1.7mm outside diameter). The borosilicate glass window material was chosen for its combination of mechanical strength and low intrinsic Raman signal (2, 3).

1. **Design Challenges:**

Several design challenges had to be overcome when redesigning the previously described forward-looking Raman device (1, 2) to the circumferential scanning device, including: 1) Maintaining a round collimated beam over long working distances; 2) minimizing back reflections from newly added components (i.e glass window) that contribute to noise; 3) maintaining a small form factor package with the ability to pass through the working channel of clinically available endoscopes; and 4) maintaining adequate signal-to-noise ratio at clinically relevant working distances and integration times to collect images at 1 rev/sec. Through the design process, many novel features have been used to simultaneously solve multiple problem areas in the performance of the system, including: a) An elliptically shaped scan mirror with a toroidal surface profile that compensates for both, beam distortion and reflections from the glass window; b) high purity fused-silica optics and deep-V antireflection coatings to reduce both intrinsic Raman scatter and back reflections within the device; and c) a unique concentrically segmented, air-spaced, doublet lens that was specifically designed to improve the collection efficiency at larger working distances.

- 1. **Collimation:**

The use of FRED simulations revealed that a non-correcting scan mirror with a flat surface allows the astigmatic focusing properties of the cylindrical window to cause one axis of the beam to expanded to double the diameter at a distance of 40 mm from the central axis of the device (**S1(a) Figure)**. At a working distance of 25 mm, the beam diameter increases to 1.58 mm along one axis, a 61% increase. At a working distance of 50 mm, the beam diameter further increases to 2.24 mm along one axis, an approximate increase of 130%. This is due to the curvature of the glass window, which effectively acts as a cylindrical lens to expand the beam along one axis. To compensate for this effect, a custom made toroidal scan mirror was fabricated such that the surface of the mirror has a concave radius of curvature (55 mm) along the minor axis of the mirror surface. This design change allows the beam to be re-collimated once it passes through the surfaces of the glass window (**S1(b) Figure)**.

The Gaussian beam focusing equation, , can be used to estimate the collimated beam diameter. *D_o_* is the collimated output beam diameter, *f* is the focal length of the collimating lens (4.63 mm), λ is the wavelength (785 nm), and *D_i_* is the mode field diameter of the single-mode 780-nm fiber (780HP, Thorlabs), which in this case is 5.0 µm. Thus, the collimated beam diameter was calculated to be 0.93 mm. Using a Gaussian model in FRED—which accounts for spherical aberration of the convex lens—predicts the 1/e^2^-intensity diameter of the collimated beam to be approximately 0.94 mm at the surface of the convex lens and 1.03 mm 60 mm from the surface of the lens due to slight diffraction of the beam. Experimentally, the collimated beam of the device was measured using a beam scanner (BeamScan, Photon) over this range of working distances. With just the convex lens in place, the profile of the beam over this range matched closely to an ideal Gaussian profile with a 1/e^2^ diameter of 0.93 to 0.98 mm over a working distance of 5 to 60 mm. Thus, the simulations matched well with the experimental results. With the mirror and window in place, using a Gaussian model in FRED predicts a collimated beam diameter of approximately 0.95 mm (x-axis = 0.95 mm, y-axis = 0.94 mm) at the surface of the window to a diameter of 1.03 mm (x-axis = 1.07 mm, y-axis = 1.01 mm) at a distance of 60 mm as measured from the central axis of the device. Experimentally, the diameter was 0.92 to 0.96 mm over a working distance of 10 to 60 mm. The x-axis varied from 0.84 to 0.89 mm and the y-axis from 0.95 to 1.05 mm over a working distance of 10 to 60 mm: nearly a 1:1 ratio.

A collimated illumination beam is advantageous in that it: 1) allows for a detection resolution that is independent of working distance, and 2) provides for a constant power density that can reliably be kept below maximum permissible exposure (MPE) limits.

- 1. **Back Reflections:**

Our FRED simulations revealed that if a 45-degree mirror were implemented in the design, approximately 1% of the incident light that reflects off both the inside and outside of the window surface is capable of reentering the multimode fibers (**S1(c) Figure**), which can be a significant noise source relative to the Raman signal. Reflected light through the multimode fibers can increase the noise floor of the signal, as we previously described (2). However, by increasing the angle of the mirror by 5 degrees, to 50 degrees, the amount of light that is back-reflected from the window surface is greatly reduced by more than 90dB (**S1(d) Figure**). Thus, as part of the custom fabrication of the mirror, a 50-degree incline was implemented in the final design to minimize the overall noise within the system. A mechanical increase in the mirror angle of 5 degrees is doubled in the optical angle; therefore, the illumination beam is incident on the inside diameter of the surface of the window at an angle of 10 degrees away from normal incidence. A majority of the first-generation rays that reflect off of the inside and outside surface of the window are reflected off the mirror at an angle and are directed away from the multi-mode fibers (black arrow in (**S1(c) Figure**). Some second-generation rays, which reflect off the inside surface of the lens holder, make it back to the surface of the multi-mode fiber, but they are ultimately rejected because they exceed the acceptable numerical aperture (NA) of the fibers. Thus, a 50-degree mirror was implemented in the final design in order to effectively illuminate back reflected light.

Both lenses have a deep-V AR coating centered at 785 nm and are separated by a precision laser-cut shim to maintain the air-glass interface. We have previously shown that such a coating reduces noise within the system (2). Also, the multi-mode fibers were stripped of their coating and surrounded by an opaque epoxy at the proximal end of the fiber bundle in order to minimize additional noise from entering the spectrometer—a design that was also implemented in our previous publication to reduce noise within the system (2).


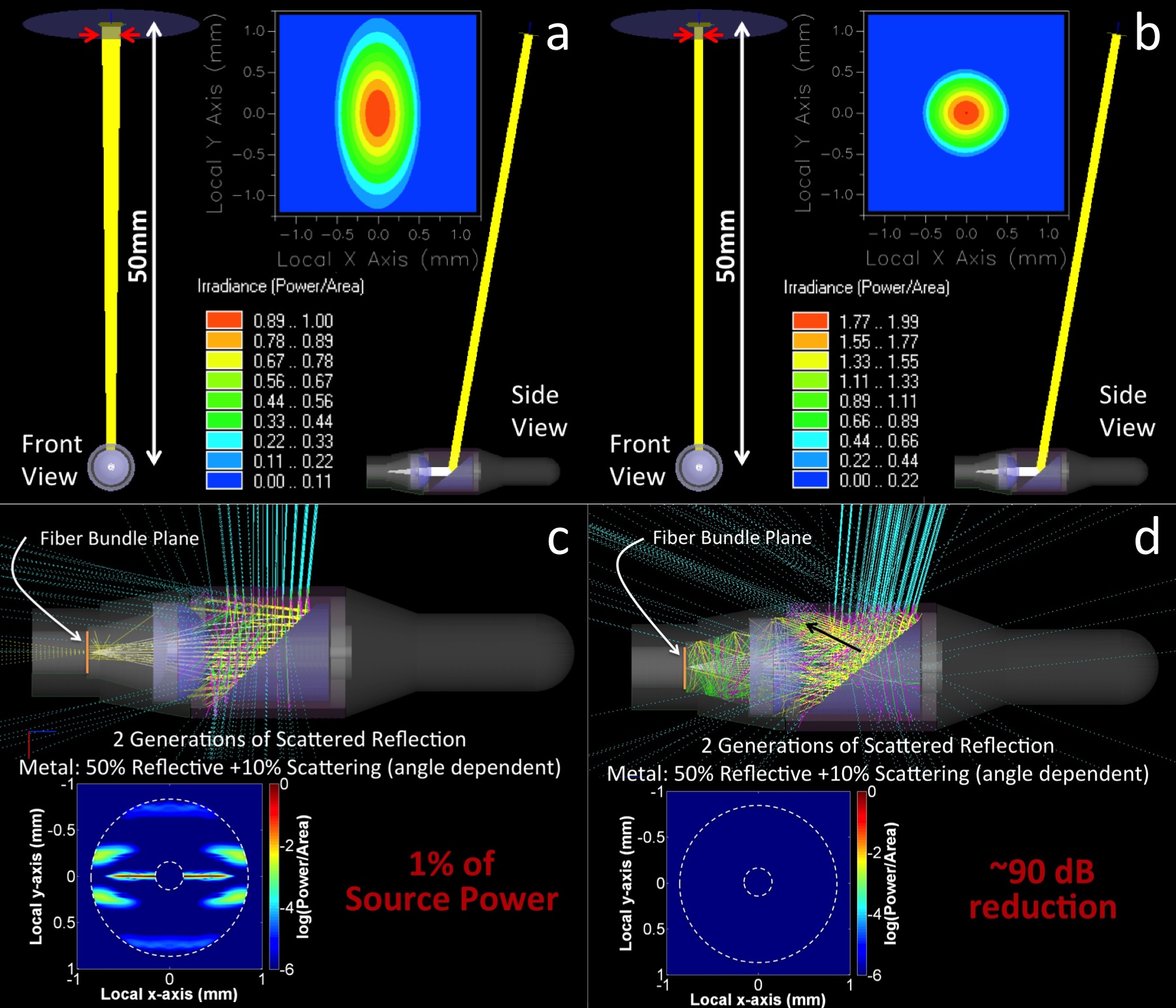


**S1 Figure.** **Optics optimization via simulation.** **(a)** Illumination beam when using a flat mirror surface. The beam expands along one axis due to the curvature of the window. **(b)** Illumination beam when using a curved mirror surface. The use of a curved mirror surface compensates for the beam divergence due to the window, resulting in a collimated beam even at a working distance of 50 mm. A mirror with a curve along its minor axis was implemented in the final design. **(c)** A 45-degree mirror results in approximately 1% of the initial power being back reflected into the multi-mode fibers of the fiber bundle. The heat map underneath represents the intensity profile at the fiber bundle plane, whose aperture is outlined by the white dashed curves. **(d)** A 50-degree mirror results in 90-dB reduction in the back-reflected power.

- 1. **Signal versus Working Distance:**
     1. **Simulations:**

As with any device requiring non-contact optical coupling of scattered light, the collection efficiency should be expected to vary with the working distance. In view of given properties of light, the 1/r^2^ free-space drop off of the maximum light collection efficiency with a fixed aperture device cannot be avoided, and thus the goals of the optical design included creating enough free parameters in the optical system to allow maximizing the collection efficiency at large working distances.

FRED software (Version 11.90.2) was used to model the optical system at the distal end of the device as well as to simulate Raman scattered light. This software was used to evaluate the collection efficiency for two different device configurations: i.) one assembly using only a single convex lens to be used for collection of the scattered light (building off the previously published forward-looking design (1, 2)); and ii.) one assembly using both a convex and concave lens to collect the scattered light.

The white rays illustrated in **S2(a) Figure** represent rays emitted from the single-mode illumination fiber at the center of the fiber bundle and are collimated by the plano-convex lens. The rays are reflected by the 50-degree elliptical mirror and pass through the wall of the borosilicate window. For analysis of back-reflections into the detector, which could be a source of noise, FRED is used to analyze the Fresnel reflections that occur at the two wall surfaces of the window. The rays are then scattered off of a scattering plane (red colored rays) in order to model a layer of SERS nanoparticles on tissue. The scattered light is given a Lambertian emission profile to model the ideal scattering of the SERS nanoparticles. Any back reflections that enter the collection fibers will be a potential noise source. The solid yellow colored rays in **S2(a) Figure** represent rays that are scattered from the scattering plane and fall within the aperture of the mirror. A subset of these rays fall within the aperture of the lens, a subset of those rays falls within the aperture of the fiber bundle (aqua colored rays), and a subset of those rays falls within the acceptance angle of each of the 36 multi-mode collection fibers.

With a single-lens configuration (i.e. no concave lens), light rays from large working distances arrive at the lens near parallel to the optic axis and are focused to the center of the fiber bundle, onto the central single-mode fiber rather than the surrounding multi-mode fibers used for collection. Therefore, the collection efficiency at 50 mm is nearly zero. To compensate for the loss of collection efficiency at the larger working distances, a second, concave lens was introduced proximal to the convex lens. This allows for significant improvement in collection efficiency at larger working distances, albeit in exchange for lower efficiency at shorter distances. At a working distance of 30 mm, the collection efficiency has a 2-fold improvement over the single lens design. At 50 mm there is a 255-fold improvement (**S2(c) Figure)**. Variations in signal with working distance are accounted for using our ratiometric analysis (2).

For each device configuration that was modeled in FRED, three scenarios were modeled: 1) no window or mirror present with fiber bundle plane modeled as an annular disk, 2) mirror and window present with fiber bundle plane modeled as an annular disk, and 3) mirror and window present with fiber bundle plane modeled as individual fibers. The purpose for modeling each scenario was to analyze how the specific geometry of the multi-mode fiber layout affects collection efficiency at varying working distances. For the simple annular disk configuration, the outside and inside diameters were 1.7 mm and 300 µm, respectively, to represent the area occupied by the multi-mode fibers (the 300-µm central region is occupied by the single-mode illumination fiber). When modeled as individual fibers, the 36 fibers were arranged in a honeycomb pattern, with each fiber occupying a 200-µm-diameter circular area—the same arrangement used for the actual device.

- - 1. **Experimental Results**

The collection efficiency over varied working distances, up to 60 mm, was evaluated and characterized using S440 nanoparticles. This experiment was repeated once for the circumferential scanning device and once for a sub-assembly of the device consisting of only the fiber bundle, lens holder, concave lens, and convex lens (i.e. no scan mirror or window) in a forward-looking, non-scan configuration. The experimental data were scaled to fit the FRED (Version 11.90.2, Photon Engineering, Tucson, AZ) simulation results such that the mean square error was minimized. The scaled fitting of the experimental data matched well with the FRED (**S2(b) Figure**).

Because this study was meant to evaluate the collection efficiency of the device with varying working distances, the SERS S440 solution used was at stock concentration (900 pM) to help ensure detection at distances as far as 60 mm. For the sub-assembly, 3 mL of S440 was place in a 9.5-cm^2^ well. At each of the working distances, nine measurements spaced 1.0 mm apart in a 3x3 grid were taken. A micrometer was used to incrementally increase the distance of the device from the sample. For the full assembly (with mirror and window), S440 was placed in a cuvette. In both cases, the output of the device was set to 40 mW, and the CCD was set to a gain of 4x with an integration time of 100 ms.

**
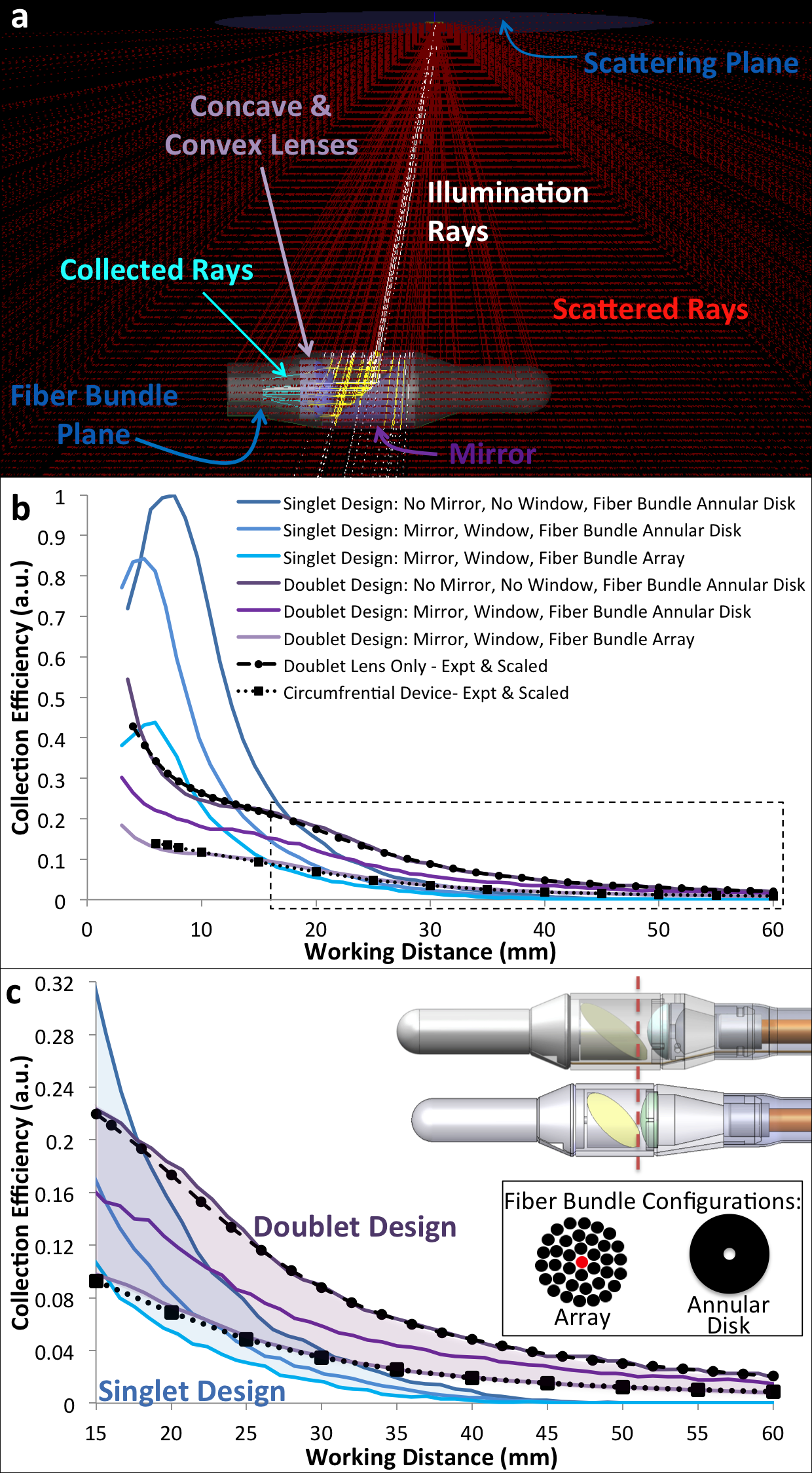
**

**S2 Figure. Collection efficiency vs. working distance analysis. (a)** FRED simulation of the distal end of the fiber bundle assembly. White rays indicate illumination rays from the single mode fiber to the scattering plane. Red rays indicate scattered rays from the scattering plane. Yellow rays indicate scattered light that is transmitted through the window and reflect off the surface of the mirror. Aqua rays indicate rays that fall within the physical aperture and numerical aperture of the multimode fibers. **(b)** Result of FRED simulations by varying the working distance of the scattering plane from the center axis of the device. The points shown are the experimentally measured Raman signal scaled to fit the FRED model. **(c)** Zoomed-in view of (b) illustrating the difference in collection efficiency between the doublet (implemented in the final design) and the singlet design.

1. **Limit of Detection at 25 mm working distance:**

SERS nanoparticles (Cabot Corporation, Boston, MA) at varying concentrations were prepared by diluting stock solution of one nanoparticle flavor (S440) with MES buffer [2-(*N*-morpholino)ethanesulfonic acid] and pipetting 3-mL samples into 9.5-cm^2^ wells. The central axis of the Raman device was placed at a fixed distance of 25 mm (the expected working distance during clinical procedures) from the surface of each of the wells. For each sample, a series of 9 acquisitions (n=9) were taken in a rectangular pattern in different locations of the well, creating a 3x3 array having 1.50-mm steps. The output power of the device was set to 68 mW, and the CCD was set to a gain of 4x with an integration time of 86 ms.

The 0.92-mm-diameter Gaussian beam has an effective area of *A_eff_* = π**D*^2^/8 = 0.33 mm^2^ and illuminates a 3.2-mm-deep column of nanoparticles (a 1.04-µL volume). At a working distance of 25 mm, the minimum detectable nanoparticle concentration was determined to be 1.25 pM, where minimum detectable signal indicates one standard deviation above background. A 1.04-µL interrogated volume at 1.25 pM corresponds to 783,000 nanoparticles. If distributed over tissue comprised of 100-µm^2^ cells, this quantity corresponds to *A_cell_/A_eff_**783,000 ~ 240 functionalized nanoparticles per cell. Thus, if 100 µm^2^ is equivalent to the cross-sectional area of approximately 9,000 SERS nanoparticles, our Raman device can detect functionalized SERS nanoparticles sparsely bound to cells and utilizing only a few hundred receptors.

International standards recognized by the FDA (per document IEC 60825-1) and the ANSI maximum permissible exposure (MPE) limit allow us to use higher power with shorter integration times; however, the power was limited by the maximum output of the laser used in our system, which yields a maximum output of 68 mW when coupled to our fiber bundle. At 68 mW, the maximum allowable integration time allowed is 86 ms, according to the ANSI and IEC limits for MPE.

1. **Paper Phantom:**

A cylindrical paper phantom was constructed to simulate an organ lumen. The axial length of the phantom was 50 mm with a diameter of 50 mm. An integration time of 10 ms/pixel was used at an axial retraction speed of 1 mm/s. The word “SPECTRA” was pipetted onto the paper using different SERS flavors (‘S’ = S493,‘P’ = S440, ‘E’ = S482, ‘C’ = S420, ‘T’ = S481, ‘R’ = S421) each at 800 pM. A dot under each letter contained at most 0.1 µL of the respective flavor. The ‘A,’ as well as the spot under the ‘A,’ was composed of a mixture of all six flavors at concentrations of 160 pM each. Spectra from a separate spot containing an equimolar mixture of 6 flavors were unmixed and used to calibrate weighting factors for each flavor. Spectral measurements from blank paper were used as a background set, and the first 10 principal components were used in the unmixing algorithm, as described previously(2).

1. ***Ex-vivo* Porcine Colon Study:**

At each injection site, 50uL of solution was injected superficially using a 29.5 gauge syringe needle. Site 1 consisted of 100 pM of all four flavors (S493, S440, S482, and S420). Site 2 consisted of 50 pM of all six flavors. Site 3 consisted of 25 pM of all six flavors. Site 4 consisted of a stepwise mixture consisting of all four flavors (S493: 100 pM, S440: 200 pM, S482: 300 pM, S420: 400 pM). Site 5 consisted of a stepwise mixture consisting of all four flavors (S493: 50 pM, S440: 100 pM, S482: 150 pM, S420: 200 pM). Site 6 consisted of a stepwise mixture consisting of all four flavors (S493: 25 pM, S440: 50 pM, S482: 75 pM, S420: 100 pM).

1. ***In-vivo* Animal Study:**

A fresh and intact pig (sus scrofa domestica) carcass was used during this study. The breed of the animal used was a Yorkshire and was a female that was roughly 6 months old in age. The animal tissue was made available through an institutional tissue sharing program. The healthy pig was euthanized as part of an acute cardiovascular imaging study that was approved by the Institutional Animal Care and Use Committee (IACUC) at Stanford University. The animal was maintained in an Association of Assessment and Accreditation of Laboratory Animal Care (AAALAC) International Accredited, Public Health Service (PHS)-assured and United States Department of Agriculture (USDA)-licensed research facility.

Nanoparticles were injected into the mucosal layer of the esophagus of a fresh and intact pig. Before injection, background spectra were obtained over a period of 50 revolutions. The output of the device was set to 68 mW with an integration time of 10 ms. Two mixtures were created: (1) an equimolar solution of S493, S440, and S420 at concentrations of 300 pM per flavor, and (2) a stepwise mixture of 150-pM S493, 300-pM S440, and 450-pM S420. 36 samples from a separate scan containing an equimolar solution was used to calibrate the weighting factor for each flavor. 500 µL of each mixture was injected in the esophageal mucosal layer of the recently euthanized pig at two different locations.

1. **Colon Phantom**

A synthetic rubber colon phantom was used in **Fig. 7** similar to the one used to train physicians. The specific colon model used was the Kyoto Kagaku Colonoscope Training Model (Kyoto Kagaku Co. Ltd, Kyoto, Japan). An 8 cm segment of the training model was used for this study. 6 different SERS flavors were pipetted onto paper and cut into circles of roughly 4 mm in diameter. 1 of these samples consisted of an equimolar solution of all 6 flavors. These samples were then placed in various axial and radial positions on the luminal surface of the colon phantom. Starting from the bottom to the top the samples were placed in the following order in roughly a helical pattern: S493, S440, S482, S420, S481, S421, and the equimolar sample. S493, S420, and the equimolar sample were placed behind a fold of the colon model. S440 and S481 were placed in front of a fold of the colon model. S482 (between fold 1 and fold 2) and S421 (between fold 2 and fold 3) were placed in relatively flat sections of the lumen. The device was retracted at a speed of 1 mm/s, the mirror had a scan rate of 1 rev/s, and an integration time of 10 ms/pix. The weighting factor from the first principal component of the background set was used to estimate working distance and generate a 3-D topographical representation of the colon phantom shown in **Fig. 7(d)**. A mean filter was implemented to reduce the pixel-to-pixel variation in calculated working distance, resulting in a smoother surface topography. Due to the limited processing power of the computer used, and the lack of a dedicated graphics card, the reconstructed image was updated on a line-by-line basis.

1. **First-In-Man Study:**

To demonstrate clinical operation of the device, it was introduced into a human volunteer through a standard colonoscope. The approving committee is the Stanford University IRB. The IRB protocol ID is 15766. Written informed consent for the study was obtained from all patients. The IRB approved written informed consent as the consent procedure. The device was sterilized and the output of the device was set to 65 mW, while the CCD was set to a gain of 4x with an integration time of 10 ms per acquisition. The motor speed was set to 1 rev/s, resulting in 100 acquisitions per revolution. Due to Food and Drug Administration (FDA) regulations, no nanoparticles were used in the study. The first 10 principal components from the acquired dataset were used as an input into the least squares algorithm. The weighting factor from the first principal component of the background set was used to estimate working distance and generate a 3-D topographical representation of the colon shown in **Fig. 8(d)**. A mean filter was implemented to reduce the pixel-to-pixel variation in calculated working distance, resulting in a smoother surface topography.

1. **Statistics:**
   1. **Limit of Detection Statistics.**

For each sample, a series of 9 acquisitions were taken in a square pattern in different locations of the well, creating a 3x3 array having 1.50-mm steps between each location. The error bars shown in **S3 Figure** are the standard deviation of the measurement (n=9) for each concentration. At concentrations of 0.57 and 1 pM, the mean signal from S440 is above the mean background (bkgd) signal. At a concentration of 1.25 pM, the mean S440 signal is over three standard deviations into the tail of the bkgd signal distribution.


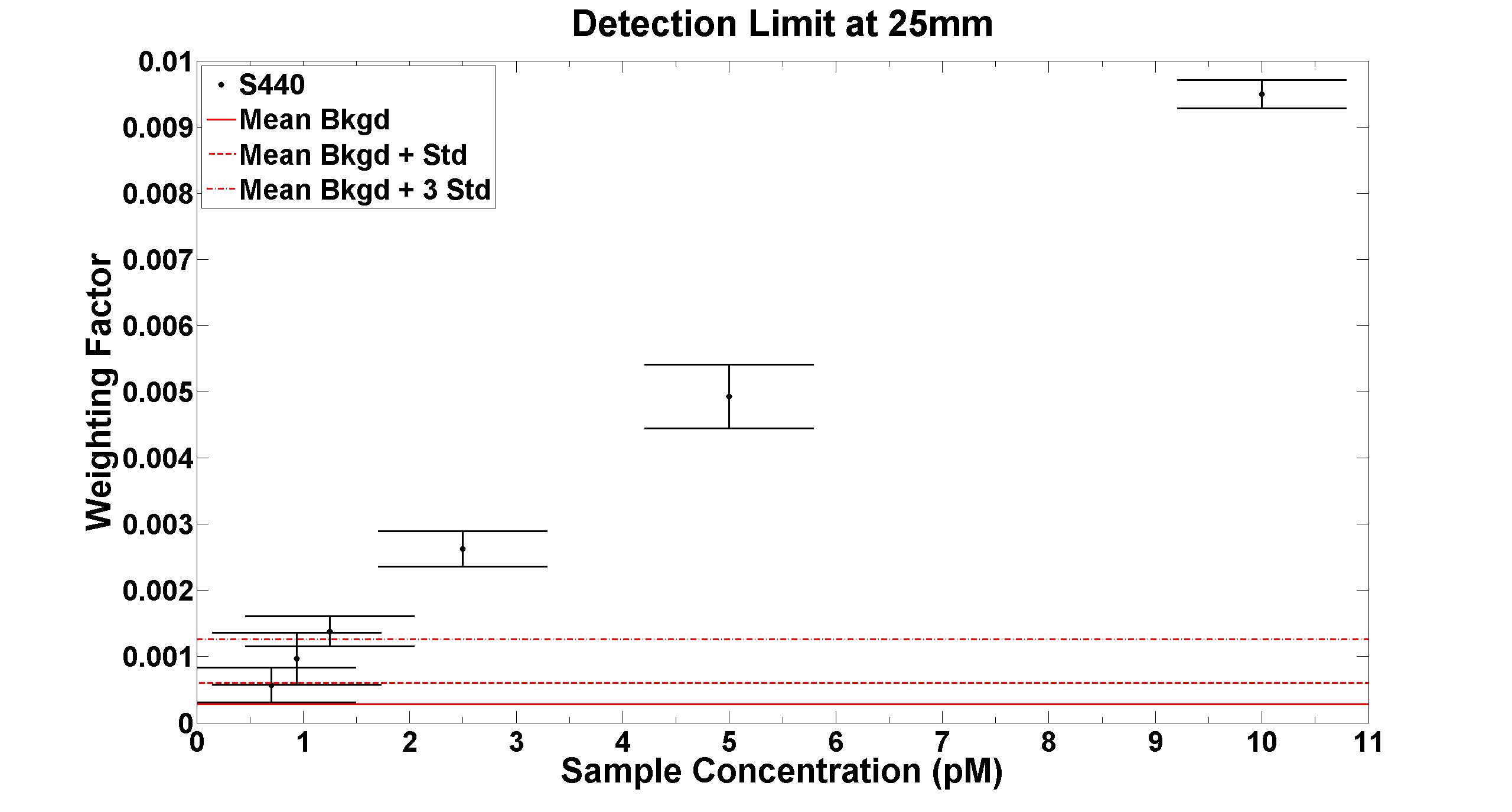


**S3 Figure.** **Limit of Detection.** Detection limit statistical analysis at a working distance of 25 mm. The center values are the average of the measurements for each concentration (n=9) and the error bars shown are the standard deviation. Also shown is the mean background signal (red), the mean background signal plus one standard deviation of the background signal (red fine dash), and the mean background signal plus three standard deviations of the background signal (red sparse dash).

- 1. **Durbin-Watson Statistical Analysis:**

The Durbin-Watson statistic tests the null hypothesis that a signal has no serial autocorrelation (i.e. adjacent signal values are unrelated) and is one strategy to quantify unmixing errors in our algorithm. If a measured Raman spectrum is perfectly defined by a linear combination of reference spectra, the residual signal after unmixing is assumed to be white noise. The Durbin-Watson statistic is a convenient indicator of first-order autoregression that is always bounded by [0, 4], regardless of the signal values or variance. The statistic is given by the following:

*N* is the number of observations and *err_t_*is the residual associated with the observation at time *t*. The residual is the error between the measured spectra and the calculated spectra. A Durbin-Watson value of 2 means zero serial autocorrelation, while values close to 0 and 4 indicate strong positive and negative correlation, respectively.

For the 7,000 acquisitions acquired during the animal study, the average Durbin-Watson value is 2.04+/-0.10 (**S4 Figure)**, suggestive of ideal unmixing. The human study consisted of 3,000 acquisitions with an average Durbin-Watson value of 2.08+/-0.10 (**S5 Figure)**. As with the animal study, there were no unmixing errors in the human study.


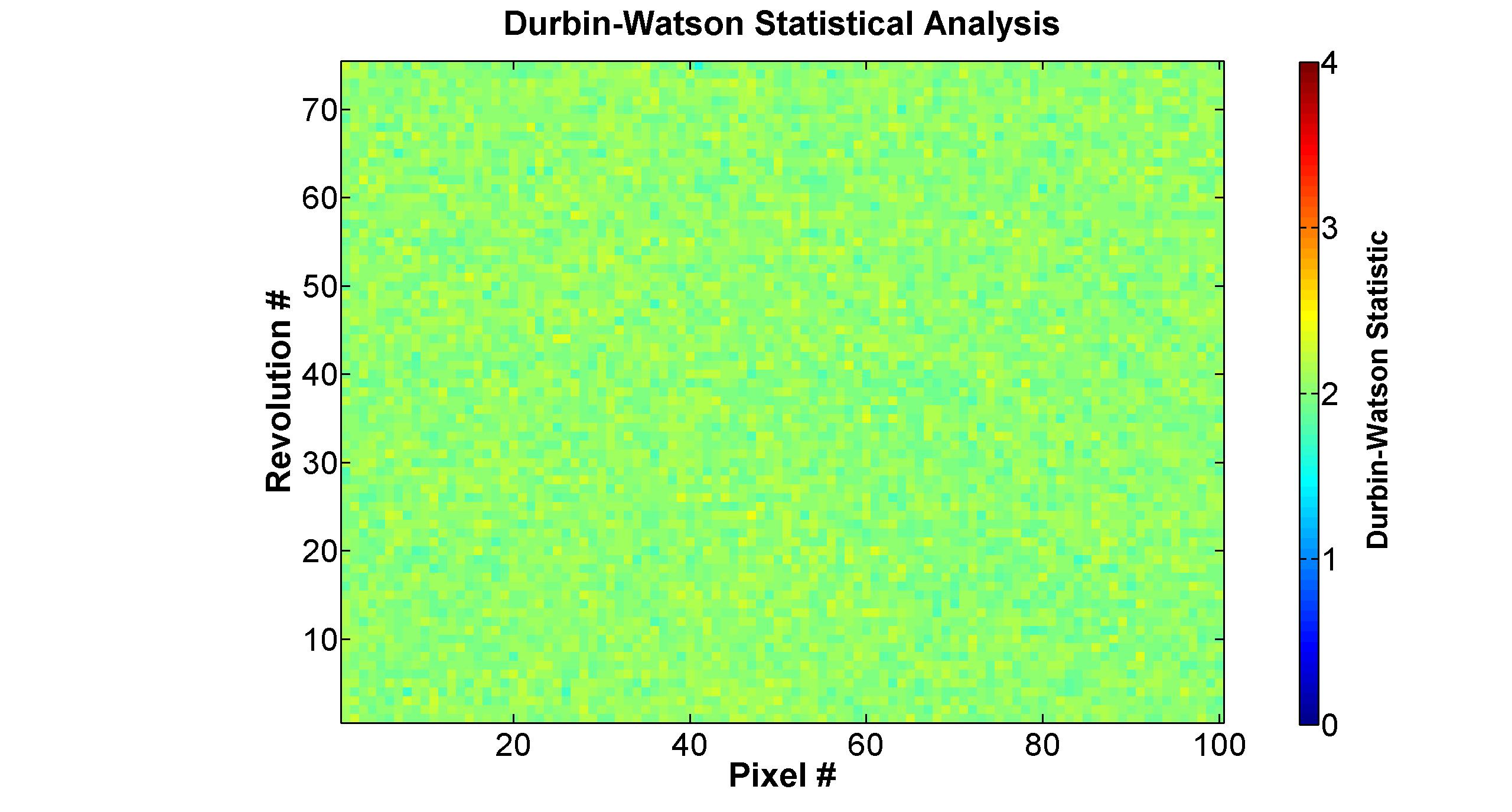


**S4 Figure.** **Durbin-Watson statistical analysis – animal study.** Durbin-Watson statistical analysis performed on the data acquired from step-wise mixture injection performed in the animal study. 70 revolutions of data were acquired for a total of 7,000 pixels with an average Durbin-Watson value is 2.04+/-0.10.


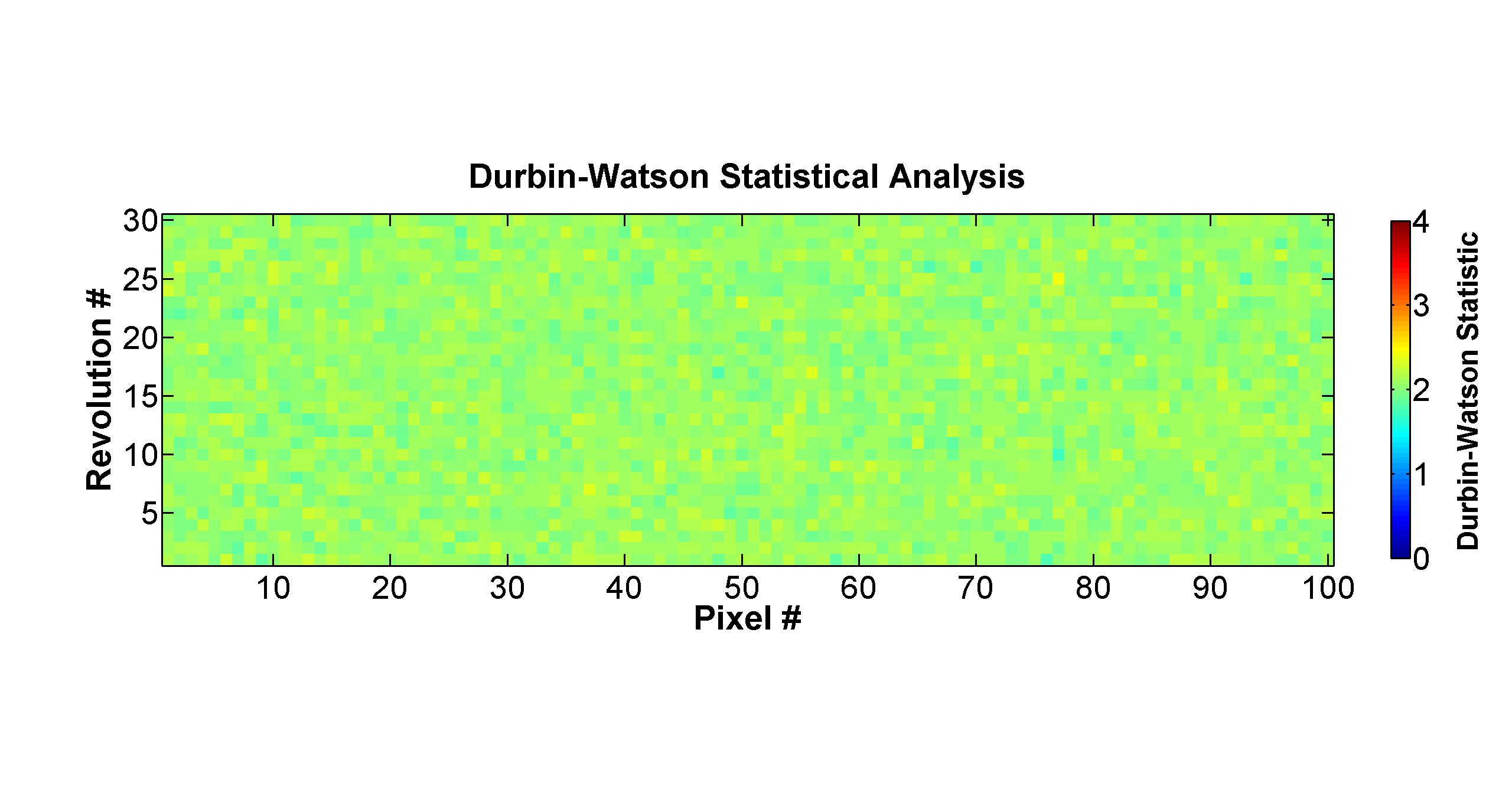


**S5 Figure.** **Durbin-Watson statistical analysis – human study.** Durbin-Watson statistical analysis performed on the data acquired from human study. 30 revolutions of data over 30 seconds were acquired for a total of 3,000 pixels with an average Durbin-Watson value of 2.08+/-0.10.

- 1. **Ratiometric Value Error Analysis.**

For the error bars shown in **Fig. 5(e)** and **Fig. 6(d)** the standard error of the mean (SEM) was used. The standard error of the mean is equal to the sample [standard deviation](http://en.wikipedia.org/wiki/Standard_deviation) divided by the square root of the sample size and is given by the following equation:

where *s* is the [sample standard deviation](http://en.wikipedia.org/wiki/Standard_deviation#Corrected_sample_standard_deviation) and *N* is the total number of samples. SEM, as opposed to standard deviation, reveals the improvement in the estimate of mean ratiometric value due to averaging of many measurements.

1. **MPE Limit Analysis.**

The American National Standards Institute (ANSI) and the International Electrotechnical Committee (IEC) Standard have set standards for maximum permissible energy (MPE) limits. The MPE limit as defined by ANSI is given by ANSI-2000 and the MPE limit as defined by IEC is given by IEC 60825-1. Both of these standards are equivalent in that the MPE limits are the same. The illumination laser of our system is operating at 785nm and the MPE limits for a laser operating between 400-1400 nm, is given by the following equation:

MPE =$1.1C_{A} t^{0.25} \left[ \frac{J}{{cm}^{2}} \right], {10}^{-7}\leq t\leq10$,

where *t* is the exposure duration in seconds and *C_A_* is the Correction Factor. For lasers operating between 700-1050 nm, the Correction Factor is given by the following equation:

$C_{A}={10}^{2(\lambda-0.700)}$,

where λ is the operating wavelength given in micro-meters. Therefore, for this application, where the Raman device is operating at 0.785 μm, the correction factor is: $C_{A}={10}^{0.170}$. Substituting the correction factor into the MPE equation: MPE =$1.1\left( {10}^{0.170} \right)t^{0.25} \left[ \frac{J}{{cm}^{2}} \right]$. Since the minimum average beam diameter outputted from the Raman device is 0.92 mm, which corresponds to a beam area of 6.64*10^-3^ cm^2^, the MPE can be simplified to

MPE = $\left( 10.8\times{10}^{-3} \right)t^{0.25} \left[ J \right]$ or MPE = 10.8$t^{-0.75} \left[ mW \right]$. For an exposer time of 10 ms, the MPE limit would be 342 mW or 51.5 W/cm^2^. Currently, the maximum power output we can achieve from our system is 68 mW or 10W/cm^2^, which is a factor of 5 less than the MPE limit.

**References:**

1. C. L. Zavaleta, E. Garai, J. T. Liu, S. Sensarn, M. J. Mandella, D. Van de Sompel, S. Friedland, J. Van Dam, C. H. Contag and S. S. Gambhir, "A Raman-based endoscopic strategy for multiplexed molecular imaging," *Proceedings of the National Academy of Sciences of the United States of America* 110(25), E2288-2297 (2013)

2. E. Garai, S. Sensarn, C. L. Zavaleta, D. Van de Sompel, N. O. Loewke, M. J. Mandella, S. S. Gambhir and C. H. Contag, "High-sensitivity, real-time, ratiometric imaging of surface-enhanced Raman scattering nanoparticles with a clinically translatable Raman endoscope device," *Journal of biomedical optics* 18(9), 096008 (2013)

3. K. B. Doyle, Mark A. Kahan, and M. A. Westborough, "Design strength of optical glass.," *Proceedings of SPIE* Vol. 5176((2003)
